# On-Road Detection of Driver Fatigue and Drowsiness during Medium-Distance Journeys

**DOI:** 10.3390/e23020135

**Published:** 2021-01-21

**Authors:** Luca Salvati, Matteo d’Amore, Anita Fiorentino, Arcangelo Pellegrino, Pasquale Sena, Francesco Villecco

**Affiliations:** 1 Department of Industrial Engineering, University of Salerno, Via Giovanni Paolo II 132, 84084 Fisciano, Italy; lsalvati@unisa.it (L.S.); apellegrino@unisa.it (A.P.); 2 Department of Pharmacy, University of Salerno, Via Giovanni Paolo II 132, 84084 Fisciano, Italy; mdamore@unisa.it (M.d.); psena@unisa.it (P.S.); 3 Pomigliano Technical Center, Fiat Chrysler Automobiles, Via Ex Aeroporto, 80038 Pomigliano d’Arco (NA), Italy; anita.fiorentino@fcagroup.com

**Keywords:** sleepiness, fatigue, driver conditions, heart rate variability, on-road experiment

## Abstract

*Background*: The detection of driver fatigue as a cause of sleepiness is a key technology capable of preventing fatal accidents. This research uses a fatigue-related sleepiness detection algorithm based on the analysis of the pulse rate variability generated by the heartbeat and validates the proposed method by comparing it with an objective indicator of sleepiness (PERCLOS). *Methods*: changes in alert conditions affect the autonomic nervous system (ANS) and therefore heart rate variability (HRV), modulated in the form of a wave and monitored to detect long-term changes in the driver’s condition using real-time control. *Results*: the performance of the algorithm was evaluated through an experiment carried out in a road vehicle. In this experiment, data was recorded by three participants during different driving sessions and their conditions of fatigue and sleepiness were documented on both a subjective and objective basis. The validation of the results through PERCLOS showed a 63% adherence to the experimental findings. *Conclusions*: the present study confirms the possibility of continuously monitoring the driver’s status through the detection of the activation/deactivation states of the ANS based on HRV. The proposed method can help prevent accidents caused by drowsiness while driving.

## 1. Introduction

One of the automotive research priorities in the development of ADAS systems is to help the driver prevent accidents. Human error is the main cause of fatalities on the road, with sleepiness or excess fatigue contributing to approximately 20–25% of all accidents [1,2] and approximately 20% of all fatal and serious accidents [3]. The terms sleepiness and fatigue are often used indistinguishably when referring to a state of decay of the driver’s condition, however they have different characteristics. Sleepiness is an intermediate state between wakefulness and sleep which has been defined as a progressive altered state of awareness associated with a desire or inclination to sleep [4]. Fatigue is considered one of the factors that can lead to drowsiness and it is a consequence of physical work or a prolonged experience and it is defined as a reluctance to continue a task [5]. Some authors distinguish fatigue from sleepiness because the former does not fluctuate rapidly, for periods of a few seconds, such as sleepiness. Usually, rest and inactivity relieve fatigue, however they make sleepiness worse [6].

One strategy for addressing this safety problem is to select the neurophysiological signals of motorists for the assessment of mental workload, fatigue and sleepiness [7], incorporating in the vehicle the function of detecting the state of the driver at all times through monitoring physical and driving performance [8].

Studies on the detection of sleepy and fatigued driving behavior have focused on two approaches: one adopts physiological signals, such as electroencephalogram (EEG) [9,10,11,12], heart rate variability (HRV) [13,14,15], the time-mediated percentage of eye closure (PERCLOS) [16,17], the facial features [18,19,20] or the behavioral characteristics (head position, sitting posture) [21,22,23,24,25] of the driver. The other approach adopts information on driving behavior and style, such as the position of the vehicle in the lane, the steering angles and the movements of the steering wheel. In turn, these methods are classified into intrusive and non-intrusive.

Approaches based on biomedical signals are particularly useful for monitoring changes in the body’s state during the sleep cycle. The information they provide goes beyond the usual systems that detect only risky circumstances (degraded driving performance or visual symptoms of lack of attention) and can potentially predict the onset of sleepiness. Changes in sleep conditions affect the autonomic nervous system (ANS) and cardiac activities [26,27,28] and HRV can be used as an indicator of the ANS’s responses to stress, sleepiness and other related factors [14,29,30,31], as well as to identify the lack of attention. A person focused on doing an activity usually displays a more regular heart rate, and as the focus on activity decreases, the heart rate becomes more irregular and HRV increases [32,33]. Chui et al. have proposed a method of detecting sleepiness based on an electrocardiogram (ECG) taken from drivers [34].

The main disadvantage of techniques based on biomedical signals is that they require the placement of sensors directly on the body: although there have already been some attempts to record them indirectly, through non-intrusive systems [35,36], they do not always manage to guarantee a continuity of the detection due to loss or degradation of physical or visual contact at the sensor/user interface. Furthermore, for safety reasons, experimental tests for the sleepiness detection are often conducted on driving simulators, in laboratories with a controlled environment and with the possibility of using measuring devices that are difficult to integrate into real vehicles. However, the main limitations of laboratory experiments are their low realism and the risk of simulator sickness [37] as well as the alteration of the spontaneous behavior of drivers: sleepiness is caused by a combination of the driver’s accumulated fatigue and boredom associated with monotonous activity, but the higher level of stimulation under real road conditions can reduce sleepiness [38]. These conditions affect the effectiveness of the experiments and therefore the development of reliable models for detecting sleepiness: for this reason, it is necessary to have a balanced and realistic quantity of detections on subjects both in periods of wakefulness and of drowsiness.

This article presents a road driving experiment with non-intrusive instrumentation, with a triple goal: (a) to collect a database of physiological signals from both wakeful and drowsy drivers, useful for studying the measurable changes related to falling attention and fatigue/sleepiness; (b) to define a continuous control index based on the long-term cardiac signal variation and compare it with the percentage of time the eyes are closed (PERCLOS) indicator for the classification of the sleepiness phases; (c) to identify patterns for the recognition of ANS activation and prevalence phases that allow us to distinguish the different phases of sleepiness and prevent it.

The percentages of time the eyes are closed for more than 70 or 80% (PERCLOS70 or PERCLOS80) are the most reliable parameters in detecting sleepiness [39,40]. Fatigued drivers show changes in visual behaviors, i.e., in the way they move their eyes or blink. It has been shown in [41] that the frequency of blinking as well as vertical eye movement increases just before sleep. Vitabile et al. used the PERCLOS parameter to evaluate the performance of their designed system under real driving conditions [42,43]. On the other hand, camera-based systems that attempt to assess the driver’s status by recognizing facial expressions only work well in ideal circumstances (head straight and face forward) [18,44,45], but they are very sensitive to glasses, head movements and lighting conditions.

## 2. Materials and Methods

### 2.1. Drowsy Driving Accident Prediction

This section describes the sleepiness detection procedure based on heart rate variability (HRV) acquired through a sensor integrated in the seat back that allows constant contact with the driver’s body [46]. The characteristics of the cardiac signal are extracted from the RRI data (interval between wave peaks) recorded while driving and the information obtained from the analysis of this signal is used in a predictive algorithm that provides the closest sleepiness/fatigue index possible to the values obtained with the PERCLOS indicator and which also manages to highlight a trend of sleepiness/fatigue phases [47,48,49,50]. In turn, the levels of sleepiness and fatigue are quantified using the Karolinska Sleepiness Scale (KSS) and the driver’s subjective estimate, respectively.

We used a universal model for the detection of sleepiness that did not take into account the individuality of the driver and that was able to provide a real-time assessment free from the need to previously acquire an identification model of sleep and wakefulness states for comparative purposes [51,52].

### 2.2. Detection System

The information relating to the heartbeat is obtained through the analysis of the acoustic pulse wave: this signal is recorded through a capacitive microphone sensor embedded in a specific support made of 3D polyester material and inserted into the seat cover. This material acts as an oscillator with a centre frequency of approximately 20 Hz and, through the phenomenon of stochastic resonance, allows both the filtering of the acoustic signal around 20 Hz for the removal of artifacts and the maximum coupling of mechanical impedance between the subject’s trunk and the sensor. This technique allows to reduce to a minimum the number of undetected beats with consequent improvement of the reliability of the analysis [53,54].

### 2.3. Analytical Model

**Figure 1:**
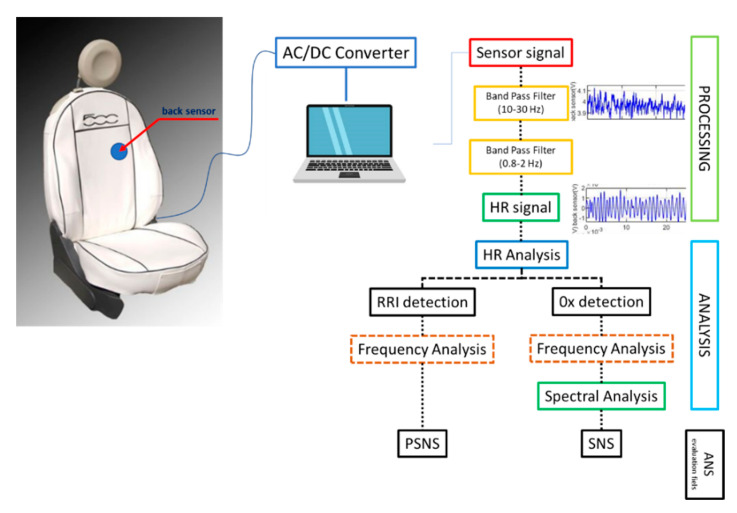
Signal processing.

The cardiac signal is filtered to eliminate the noise components due to respiration or movement artifacts: the information on the vibration due to the heartbeat is contained in signals around 20 Hz [53,54] which are therefore passband filtered from 10 to 30 Hz. In order for this signal to take on the wave form of the cardiac cycle (having a frequency close to 1 Hz), it is further passband filtered between 0.8 and 2 Hz (Figure 1). A second-order Butterworth IIR bandpass filter is applied.

**Figure 2:**
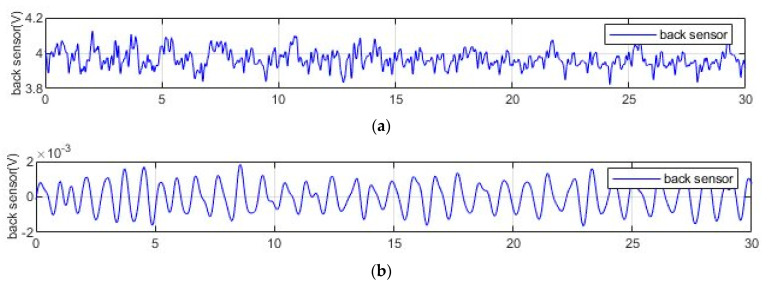
30-s heartbeat signal: before (**a**) and after (**b**) processing.

**Figure 3:**
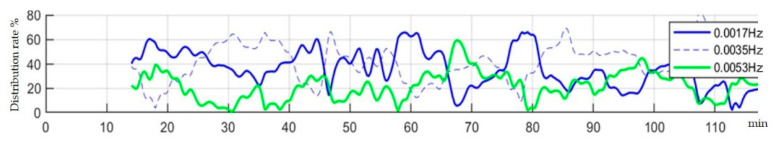
Example of spectral distribution obtained from one of the subjects.

The waveform thus obtained (Figure 2) allows us to evaluate two types of interval: the one between the peaks and the variability of which in terms of average frequency detected in constant time segments we believe is useful for identifying the prevalence states of the parasympathetic nervous system (PSNS) and therefore any conditions of exhaustion; and the interval between the points of intersection with the zero baseline whose spectral analysis (Figure 3) is used to estimate a decrease in sympathetic nervous activity (SNS), i.e., a reduced level of arousal linked to the occurrence of drowsiness episodes [55,56,57].

The intervals between peaks and between zero-line crossing points are first converted into the corresponding frequencies and these, in turn, are averaged over a 5 s interval. This technique allows to produce an output even if the sensor fails to pick up the cardiac signal.

In order to determine the fatigue state, it is necessary to evaluate the changes in the human condition in long-term cycles that can be highlighted by analyzing the ultra-low frequency components of HRV [58]. Similarly, human homeostasis manifests itself through fluctuations in an ultra-low frequency band: the region between 0.001 and 0.007 Hz is believed to contain information relating to long-term regulation [59,60], in particular there are fluctuations in the frequency band around 0.0017 Hz (whose signal is associated with a decrease in sympathetic activity), 0.0035 Hz (associated with the evolution of the fatigue level) and 0.0053 (it expresses the level of control exerted by the SNS during an activity).

From the analysis of the frequency trend of the intersection points with the zero line, assuming 100 the total values of the power spectra of the aforementioned frequency components, a spectral distribution with three waveforms is obtained (Figure 3). Taking into consideration the results relating to the tests in which episodes of drowsiness occurred, by examining the graphs of the percentage distribution frequency, it is observed that in the imminence of the periods in which drowsiness or falling asleep is reported occurs the condition:

$$\frac{\theta +\alpha }{\beta }>2\theta ,\;\alpha >\beta$$

and more precisely it is possible to associate the sleep state to the case in which θ>α and the drowsiness state to the case in which α>θ. In the case α≫θ, with ascending *α* and *θ* steady, it can be assumed that there is fatigue of the subject. The increase in the distribution of α (ascending) compared to θ (descending) can mean that the organism tries to resist sleep, while the increase in β can indicate the resumption of an activation state because, in cases where no onset of drowsiness occurred, this distribution has concentrated peaks in the 20–60% range, while in cases where drowsiness occurred this distribution had concentrated peaks in the 0–30% range.

### 2.4. Drowsiness Scale

KSS is a self-signaling method in which the driver is asked every 15 min to provide a number between 1 and 9 of their sleepiness level (1 indicates fully aware and 9 indicates a very sleepy condition) [44]. However, this method has two limitations: it is unable to continuously monitor the driver’s sleepiness and it is based on self-assessment, while based on our experiments drivers often do not have a precise idea of their level of sleepiness. To reduce this negative effect, a camera was installed on the car’s dashboard in order to capture the driver’s face during his performance and to be able to evaluate the PERCLOS index. In calculating this parameter, we took into account any manifestation of drowsiness (70% of partial closing of the eyelids, blinking, yawning). The detection was divided into “*i*” frames of 10 s, giving each of them the value 1 in the event of drowsiness and the value 0 in the opposite case. The PERCLOS index was evaluated every minute as an average of 6 frames:

$$\mathrm{PERCLOS}=\frac{\sum_{i=1}^{6}Drowsiness\;event\;\left[i\right]}{6}\times 100\;\left(\%\right)$$

where by “Drowsiness event” we mean the occurrence of any of the three manifestation of drowsiness aforementioned.

In this way, six ranges of values were identified [0–0.33, 0.34–066, 0.67–1], grouped two by two so as to distinguish three different macro areas: alertness, hypovigilance and sleepiness. To take into account the effect of fatigue on the conditions of sleepiness and the weight of the subjective judgment which corresponded to a more or less high level of fatigue at each level of sleepiness, a scale was also defined for the algorithm used with values 0 to 12 that could adjust to the levels of sleepiness and fatigue expressed with the KSS (Table 1).

## 3. Results

### 3.1. Experiments

#### 3.1.1. Experimental Environment

**Figure 4:**
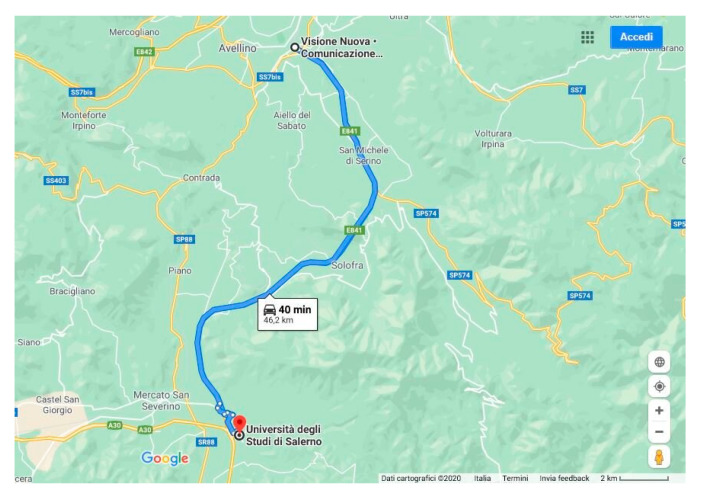
Test course.

The tests were carried out on a motorway circuit 46.2 km long (Figure 4), which was run twice in the afternoon to allow for smooth traffic conditions. The total time taken is approximately 1 h 35 m. The speed limit was imposed at 80 km/h. The car used is a city car without driver assistance systems and equipped with a seat cover containing the microsensor for detecting the heart signal.

#### 3.1.2. Experiment Subjects

Three drivers over the age of 30 performed this experiment for a total of 14 sessions. They are all healthy males with decades of driving experience. FCA professional drivers were recruited and test were performed during routine workday’s activities, with all participants that received an explanation about the purpose of test and signed their informed consent. The subjects were asked to sleep at least 7 h the night before the driving experiment, not to smoke and not to take caffeine, alcohol or energy drinks after waking up until the time of the experiment. This preparation allows to replicate in a more realistic way natural conditions of fatigue and postprandial sleepiness. Before starting the test, each driver adjusted the backrest so that his back was naturally in contact with the seat: this action allowed for constant body contact with the seat cover.

#### 3.1.3. Data Acquisition

Driving information is collected on the car in real-time, including the heart signal at a sampling rate of 200 Hz and the video recording of the driver’s face, who is also administered a self-assessment questionnaire of their drowsiness and fatigue. The videos with the driver’s oral assessment every 15 min help to estimate the driving status and create a corresponding set of learning data useful for defining the level scale used as the output of the algorithm.

#### 3.1.4. Experimental Procedure

The driving experiment consisted in completing the path outlined twice without stopping so that the conditions of the participants were monitored for at least an hour and a half and they could be in a more realistic state of sleepy/fatigued driving. Drivers reported their feelings on the degree of sleepiness/fatigue every 15 min according to the KSS scale.

### 3.2. Experimental Results

The data relating to the individual experiments were evaluated independently and not aggregated by subject or time of execution, so as to be able to analyze the performance in an absolutely random manner and obtain an analysis independent of the subjectivity of each driver’s conditions (health, emotional, sleep, fatigue). Since the PERCLOS index is calculated every minute while that of the algorithm provides a result every 18 s, it was decided to evaluate the correspondence of the levels to which the numerical indices belong every 5 min for a total of 275 detections distributed in the 14 tests performed.

**Figure 5:**
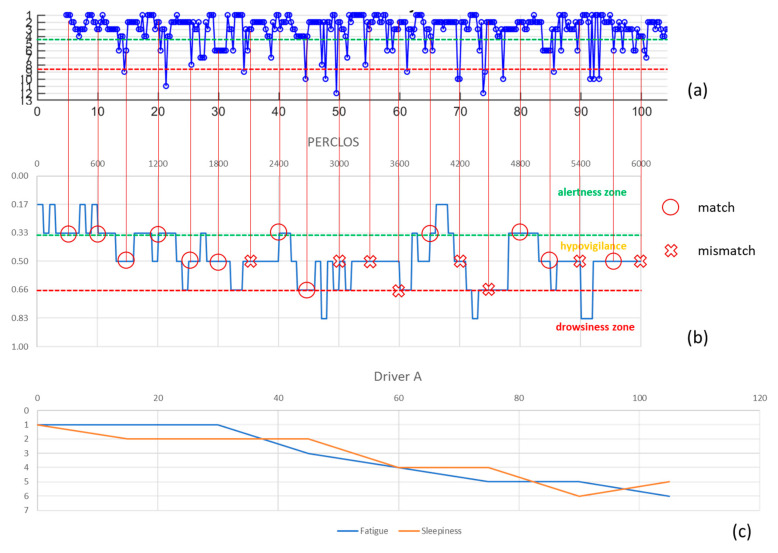
Driver A’s detections: (**a**) algorithm score, (**b**) PERCLOS index, (**c**) subjective evaluation.

**Figure 6:**
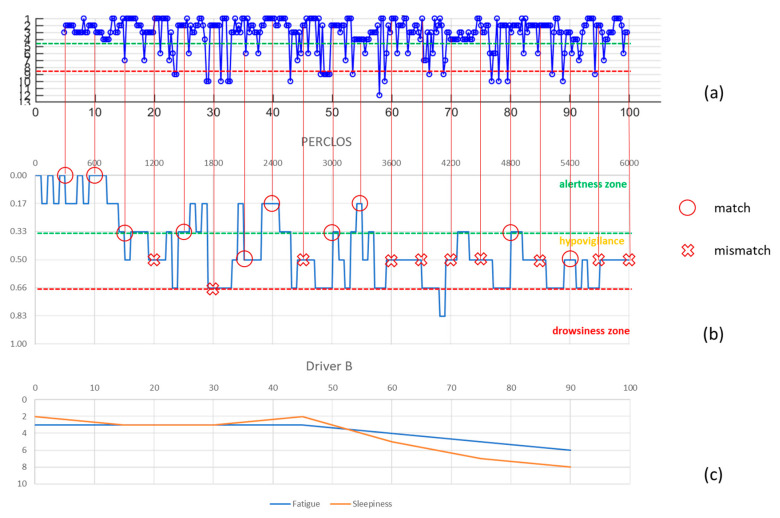
Driver B’s detections: (**a**) algorithm score, (**b**) PERCLOS index, (**c**) subjective evaluation.

**Figure 7:**
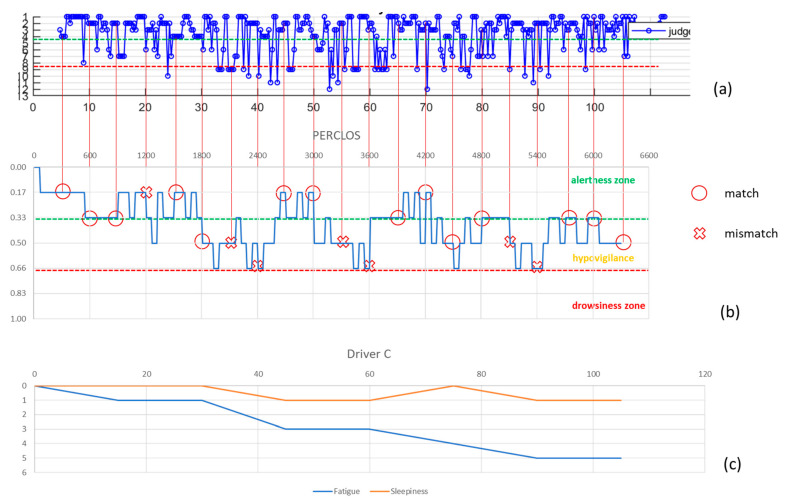
Driver C’s detections: (**a**) algorithm score, (**b**) PERCLOS index, (**c**) subjective evaluation.

For explanatory purposes, Figure 5, Figure 6 and Figure 7 show the cases relating to two of the participating subjects: in (a) the graph of the values resulting from the algorithm used for the assessment of sleepiness, in (b) the PERCLOS index and in (c) the driver’s subjective assessment are shown. In each graph, the three macro-areas that had been defined in the sleepiness scale were distinguished and to evaluate the accuracy of the algorithm it was considered that there was a correspondence of the result in the case in which the values of the PERCLOS and of the index calculated with the algorithm fell into the same macro-area. Therefore, the accuracy thus calculated is 63%. The predictive effectiveness of the algorithm was also assessed by considering, in correspondence with each of the 275 detection events, the trend of the scores arising in the 5 min or 90 s prior to the evaluation of PERCLOS: respectively 16 and 5 scores were considered and the macro area was attributed to the trend according to where the relative majority of its points fall. The accuracy was respectively 44% and 56% (Table 2) highlighting that, given the considerable variability in the measurement of the trend of fatigue and related sleepiness, the algorithm is less efficient when a history of previous values is incorporated, but more accurate when taking into account values closest to the chosen detection point.

## 4. Discussion

Comparing the self-assessment graphs of sleepiness/fatigue with the PERCLOS index it is evident that, on the one hand, the driver’s state does not necessarily reflect in expressive or behavioral manifestations typical of a stressed condition, on the other hand the driver does not have a clear perception of his state enough to be able to quantify it. However, the subject’s judgment was useful in defining a drowsiness scale in which the effect of fatigue could be taken into account.

From the comparison of PERCLOS with the results of the used model, it is observed how the evaluation of the driver’s status shows similar trends, although failing to ensure high accuracy at all times, especially in the transition phases which turn out to be the cause of error in 82% of cases.

The drowsiness detection model described is sufficiently adherent to the state of the monitored drivers, at least as long as they are not in limit conditions. The difficulty of carrying out tests on real vehicles to detect the conditions of the driver in a state of sleep deprivation is one of the main limitations to experimentation.

## 5. Conclusions

In this study, the potential of an algorithm for identifying the driver’s sleepiness/fatigue states was evaluated. This was achieved by analyzing the corresponding physiological signals, from the eyes and the heart.

The impossibility of carrying out a further variability analysis between a single subject’s data or across subjects, due to the different and changing traffic conditions which in turn influence the behavior and physiological states of the driver, is a limitation for accuracy assessment which can be overcome by intensifying the number of tests in an environment that is as controlled as possible.

The obtained results have not currently exceeded the accuracy of feature extraction methods based on the detection of blinking, facial expressions or steering angle. However, this system overcomes some of the limitations of these techniques as it allows continuous and contactless monitoring, limits interference related to environmental factors (brightness, bumpy paths) or contingent ones (use of glasses, head movements) and makes use of devices without contraindications.

Being able to perform tests of longer duration and in more critical conditions for the subjects would allow us to know how the physiological conditions change in the state of transition from wakefulness to sleep and how the subject manifests his opposition to physical failure.

## Figures and Tables

**Table 1:** Drowsiness level description.

Karolinska Sleepiness Scale			Post-Processed Sleepiness Scale	
Level	Verbal Description	Vigilance Stage	Level	Verbal Description
1	extremely alert	alertness	0	negligible signs of fatigue
2	very alert		1	
			2	
3	alert		3	
			4	
4	rather alert	hypovigilance	5	slight fatigue
5	neither alert nor sleepy		6	
6	some signs of sleepiness		7	
			8	
7	sleepy, but no effort to keep alert	drowsiness	9	increasing fatigue and drowsiness
8	sleepy, some effort to keep alert		10	
			11	slight drowsiness
9	extremely sleepy, fighting sleep		12	strong drowsiness

**Table 2:** Accuracy indices.

Driver	Test	n° of Detections	Match	Matching with the Last 5-min Trend of Scores	Matching with the Last 90-s Trend of Scores
A	1	19	13	9	12
	2	18	11	7	11
	3	20	12	9	9
	4	21	14	10	12
	5	20	12	8	13
B	1	18	12	9	10
	2	19	13	9	12
	3	18	12	7	12
	4	18	12	8	10
	5	19	10	6	7
C	1	22	13	9	10
	2	22	12	8	8
	3	21	14	9	11
	4	20	12	7	9
tot	14	275	172	115	146
Success %			63	44	56
